# USP28 facilitates pancreatic cancer progression through activation of Wnt/β-catenin pathway via stabilising FOXM1

**DOI:** 10.1038/s41419-021-04163-z

**Published:** 2021-09-28

**Authors:** Leifeng Chen, Zheng Xu, Qing Li, Qian Feng, Cihua Zheng, Yunyan Du, Rongfa Yuan, Xiaogang Peng

**Affiliations:** 1 Department of General Surgery, the Second Affiliated Hospital of Nanchang University, Nanchang, 330006 Jiangxi Province China; 2 Cancer Center, Renmin Hospital of Wuhan University, Wuhan, 430060 Hubei China; 3 Department of Pathology, the Second Affiliated Hospital of Nanchang University, Nanchang, 330006 Jiangxi Province China; 4 Department of Medical, Jiangxi Provincial People’s Hospital of Nanchang University, Nanchang, 330006 China; 5 Jiangxi Province Key Laboratory of Molecular Medicine, the Second Affiliated Hospital of Nanchang University, Nanchang, 330006 Jiangxi Province China

**Keywords:** Pancreatic cancer, Cell growth

## Abstract

Ubiquitination is an important post-translational modification that can be reversed by a family of enzymes called deubiquitinating enzymes (DUBs). Ubiquitin-specific protease 28 (USP28), a member of the DUBs family, functions as a potential tumour promoter in various cancers. However, the biological function and clinical significance of USP28 in pancreatic cancer (PC) are still unclear. Here, we showed that PC tumours had higher USP28 expression compared with that of normal pancreatic tissues, and high USP28 level was significantly correlated with malignant phenotype and shorter survival in patients with PC. Overexpression of USP28 accelerated PC cell growth, whereas USP28 knockdown impaired PC cell growth both in vitro and in vivo. Further, we found that USP28 promoted PC cell growth by facilitating cell cycle progression and inhibiting apoptosis. Mechanistically, USP28 deubiquitinated and stabilised FOXM1, a critical mediator of Wnt/β-catenin signalling. USP28-mediated stabilisation of FOXM1 significantly promoted nucleus β-catenin trans-activation, which in turn led to the activation of the Wnt/β-catenin pathway. Finally, restoration of FOXM1 expression abolished the anti-tumour effects of USP28-silencing. Thus, USP28 contributes to PC pathogenesis through enhancing the FOXM1-mediated Wnt/β-catenin signalling, and could be a potential diagnostic and therapeutic target for PC cases.

## Background

Pancreatic cancer (PC) is the seventh leading cause of cancer-related deaths worldwide [1]. Its poor prognosis is reflected by a 5-year survival rate around 8%, mainly due to late diagnosis and lack of effective therapies [2–4]. Therefore, better understanding of the molecular mechanisms involved in PC progression is an urgent need to identify novel targets and develop new therapeutic strategies for treating PC.

Alterations in protein ubiquitination are commonly associated with cancer. Deubiquitinating enzymes (DUBs) counteract the activities of E3 ligases, and are implicated as important molecules that modulate ubiquitination [5, 6]. As a result, they have emerged as promising therapeutic targets for cancer treatment [7]. Ubiquitin-specific protease 28 (USP28), a member of the DUBs family, is involved in physiological processes of cell proliferation, differentiation, apoptosis, DNA damage repair, and stress response [8–10]. Recent studies have shown that USP28 is highly up-regulated and is correlated with poor clinical prognosis in a variety of cancers, such as colon cancer [11], non-small cell lung cancer [12], and bladder cancer [13]. Because of its deubiquitinase activity, USP28 interacts with Myc to catalyse the deubiquitination of Myc, thereby promoting its stabilisation and contributing to tumour cell growth in colon and breast carcinomas [14]. However, the biological functions and expression patterns of USP28 in PC are not known.

FOXM1 (forkhead box protein M1), as a key proliferation-associated transcription factor, which maintains cancer hallmarks by activating the expression of target genes at the transcriptional level [15, 16]. Elevated FOXM1 expression is observed in a variety of human cancers, and inhibition of FOXM1 significantly suppresses the malignant phenotype of cancer cells [17, 18]. Importantly, FOXM1 is a critical regulator of cell cycle progression, and multiple studies have demonstrated that it plays critical roles in PC growth as well [19–21]. Therefore, uncovering the regulatory mechanisms of FOXM1 will provide new insights into the pathogenesis of PC as well as new therapeutic strategies against this deadly cancer.

Thus, in this study, we aimed to identify the significance of USP28 and FOXM1 in PC development, and investigated PC progression via the USP28/FOXM1/β-catenin axis.

## Methods

### Patients and tumour specimens

Matched cancerous and normal pancreatic tissue specimens were obtained from 102 patients with PC admitted to the Department of General Surgery, the Second Affiliated Hospital of Nanchang University from 2015 to 2019. The specimens were immediately snap-frozen in liquid nitrogen, and stored at −80 °C for further analysis. Written informed consent was obtained from all patients, and the research procedure was approved by the Ethics and Research Committees of the Second Affiliated Hospital of Nanchang University. The clinical characteristics of all patients are summarised in Table 1.

**Table 1:** Relationship between USP28 expression and clinicopathological features.

Parameters	*n*	USP28 expression		*P* value
		Low (*n* = 34)	High (*n* = 68)	
Age (years)				*P* = 0.777
≤65	44	14	30	
>65	58	20	38	
Sex				*P* = *0.483*
Female	49	18	31	
Male	53	16	37	
Tumour size (cm)				***P*** = ***0.017***
<5	68	28	40	
≥5	34	6	28	
TNM stage				***P*** < ***0.001***
T1–T2	42	24	18	
T3–T4	60	10	50	
Distant metastasis				*P* = *0.152*
No	70	27	43	
Yes	32	7	25	
Histologic grade				*P* = *0.223*
High	31	13	18	
Low to medium	71	21	50	
Differentiation				***P*** < ***0.001***
Well	37	29	8	
Moderate/poor	65	5	60	

Bold Italic values are statistically signifcant, *p* < 0.01.

### Cell lines and cell culture

Normal pancreatic ductal epithelial cell line (H6C7; served as the control) and PC cell lines (AsPC-1, PANC-1, BxPC-3 and SW1990) were purchased from American Type Culture Collection (Manassas, VA, USA). The cell lines were cultured in Dulbecco’s modified Eagle’s medium (Gibco, USA) supplemented with 10% foetal bovine serum and 100 units/mL of penicillin-streptomycin (Invitrogen, USA) at 37 °C in a humidified atmosphere containing 5% CO_2_.

### Quantitative real-time (qRT)-PCR

Total RNA was isolated from cells using Trizol reagent (Invitrogen, USA). qRT-PCR was performed using SYBR Green qPCR Master Mix (Clontech Laboratories, USA) with Applied Biosystems® 7900HT Fast Real-Time PCR System (Thermo Fisher Scientific, USA). The primers used are followed: USP28, 5′-ACTCAGACTATTGAACAGATGTACTGC-3′ and 5′-CTGCATGCAAGCGATAAGG-3′; FOXM1, 5′-ACCGCTACTTGACATTGGAC-3′ and 5′-GGGAGTTCGGTTTTGATGGTC-3′; GAPDH, 5′-CATACCAGGAAATGAGCTTGAC-3′ and 5′-AACAGCGACACCCACTCCTC-3′.

### Western blot, immunohistochemistry (IHC) and immunofluorescent (IF) assay

For western blot assay, equal amounts of cell lysates were resolved by SDS/PAGE, electrotransferred to polyvinylidene fluoride (Millipore, USA) membranes and blocked in 5% skim milk. Membranes were immunoblotted with the indicated primary antibodies, followed by the appropriate HRP-conjugated anti-mouse/rabbit (KPL) or anti-goat (Beijing CoWin Biotech, China) secondary antibodies. Immunoreactive bands were visualized with chemiluminescence kits chemiluminescence (Pierce, USA). The following antibodies were used: antibodies against USP28 (1:1000, Abcam, ab126604), cyclin D1 (1:1000, Cell Signaling, 55506), CDK4 (1:1000, Cell Signaling, 12790), PCNA (1:1000, Cell Signaling, 13110), Cleaved Caspase-3 (1:1000, Abcam, ab2302), Caspase-3 (1:1000, Abcam, ab13847), Cleaved PARP (1:1000, Cell Signaling, 5625), PARP (1:1000, Cell Signaling, 9532), β-catenin (1:1000, Abcam, ab32572), c-Myc (1:1000, Abcam, ab32072), VEGF (1:500, Santa Cruz, sc-7269), Survivin (1:1000, Abcam, ab76424), Flag (1:1000, Sigma, F1804), HA (1:1000, Abmart, M20003), FOXM1 (1:1000, Cell Signaling, 5436), ubiquitin (1:1000, Enzo Life Sciences, PW8805) and GAPDH (1:1000, Abcam, ab8245).

For IHC staining, the matched cancerous and normal pancreatic tissue samples were fixed, embedded, sectioned, and deparaffinized. Then, the sections were blocked using serum-free protein block buffer (DAKO, USA) for 30 min, after which they were incubated with anti-USP28 (1:200, Abcam, ab126604), anti-FOXM1 (1:200, Cell Signaling, 5346) anti-Cleaved Caspase-3 (1:200, Abcam, ab2302) and anti-Ki67 (1:200, Cell Signaling, 2586). For IF assays, after the indicated treatments, the cells were fixed with 4% paraformaldehyde (PFA) and incubated with 0.1% TritonX-100, then stained with anti-β-catenin (1:100, Abcam, ab32572), anti-USP28USP28 (1:100, Abcam, ab126604) and anti-FOXM1 (1:100, Cell Signaling, 5436). After incubated secondary antibodies, the cell nuclei were counterstained with DAPI. Then, cells were viewed using a laser-scanning confocal microscope (Zeiss, Germany). Two different pathologists evaluated all the specimens in a blinded manner.

### Overexpression constructs, shRNA plasmids and cell transfection

Eukaryotic expression vectors encoding either USP28 or FOXM1, and plasmids encoding short hairpin RNAs (shRNAs) against either USP28 or FOXM1 were synthesised by GenePharma (Shanghai, China). Then, the PC cells were transfected with these overexpression constructs or shRNA plasmids using Lipofectamine 3000 (Invitrogen, USA) according to the manufacturer’s instructions. Lastly, G418 was used to screen and establish PC cell lines stably transfected with USP28-overexpression vector or USP28 shRNA plasmid.

### Cell proliferation and colony formation assays

For the cell proliferation assay, PC cells were cultured in 96-well plates (1000 cells per well; experiment performed in triplicate). At the indicated time points, viable cells were tested using Cell Counting Kit-8 (CCK-8) according to the manufacturer’s instructions. For the colony formation assay, PC cells that were transfected with overexpression constructs or shRNA plasmids were selected after 4–6 d of culture and then plated in a 6-well plate (2000 cells per well). Finally, after 2–3 weeks of culture, the cells were incubated with fixation buffer (5% acetic acid and 5% methanol) followed by staining with 0.5% crystal violet solution.

### Flow cytometry assay

For the cell cycle assay, the PC cells were harvested at exponential growth phase, and single-cell suspensions containing 1 × 10^5^ cells were fixed with 70% alcohol. Then, the cell cycle was monitored using propidium iodide staining and detected with a FACScan flow cytometer (BD Biosciences, USA). For the cell apoptosis assay, early and late apoptotic cells were evaluated by Annexin-V Fluorescein Isothiocyanate and Propidium Iodide Apoptosis Detection Kit (BD Biosciences, USA) according to the manufacturer’s instructions. Finally, the stained cells were analysed with a FACScan flow cytometer.

### Tumorigenicity assay and bioluminescence imaging

PC cells used for injection were stably transduced with firefly luciferase gene and enabled regular in vivo monitoring of tumour growth by bioluminescent imaging. For in vivo tumorigenicity assays (1 × 10^6^ in 100 ml PBS) were injected subcutaneously into the flanks of nude mice (male BALB/c-nu/nu, 6–8 weeks old). Then, for in vivo signal detection, the mice were anesthetized with isofluorane and then imaged in a Lumina Series III IVIS (In Vivo Imaging System) instrument (PerkinElmer, MA, USA). The animal work was approved by the Ethics Committee for Animal Experiments of the Second Affiliated Hospital of Nanchang University.

### Luciferase reporter activity analysis

PC cells (1 × 10^5^ cells per well) were co-transfected with TOPFlash luciferase reporter plasmid (used to assess the transcriptional activity of β-catenin), pRL-CMV vector, and either the shUSP28 plasmid or USP28 vector using Lipofectamine 3000 transfection reagent for the overexpression study. The cells were harvested at 48 h post-transfection and luciferase activity was quantified by a Dual-Luciferase Reporter Assay System (Promega, USA).

For treatment with exogenous Wnt3a, the recombinant human Wnt3a (R&D Systems, 5036-WN) was reconstituted in sterile PBS containing 0.1% FBS. Medium containing 150 ng/ml of recombinant human Wnt3a was added to cell cultures.

### LC-MS/MS analyses

LC-MS/MS Analyses was performed as described previously [22]. USP28-silenced PC cells and control cells were analysed by Tandem Mass Tag (TMT) labelling and LC-MS/MS. Whole-cell lysates were extracted from these cells using lysis buffer with 1% proteinase inhibitor cocktail (Sigma, USA), and quantified by bicinchoninic acid method. Equal amounts of protein from the PC and control cells (100 μg) were reduced, alkylated, and then precipitated with acetone. The precipitates were then resuspended in 200 mM tetraethylammonium bromide and digested with trypsin. The obtained samples were then combined, separated by high-performance liquid chromatography, and analysed by LC-MS/MS.

### Co-immunoprecipitation (Co-IP) and in vivo ubiquitination assay

For Co-IP assay, the cell lysates were incubated with a specific primary antibody overnight at 4 °C, followed by incubation with protein A/G-Sepharose beads (Santa Cruz, USA). Then, the co-precipitated proteins were collected and identified by immunoblotting analysis against specific antibodies. For the in vivo ubiquitination assay, the cells were co-transfected with the previously mentioned plasmids. After transfection, the cells were treated with 50 μg/mL of proteasome inhibitor MG132 for 12 h. Finally, the cells were lysed for western blotting assay and immunoprecipitation by following the same protocol as that of the Co-IP assay.

### Statistical analysis

All data are expressed as mean ± standard error of mean by using GraphPad Prism 6 (GraphPad Software, USA). Significant differences were analysed using Student’s *t*-test and two-tailed distributions. Kaplan–Meier method was used to calculate the survival curve, and log-rank test was used to determine the significance. *P* < 0.05 was considered to be significant.

## Results

### USP28 was overexpressed in human PC specimens and associated with poor prognosis

**Fig. 1:**
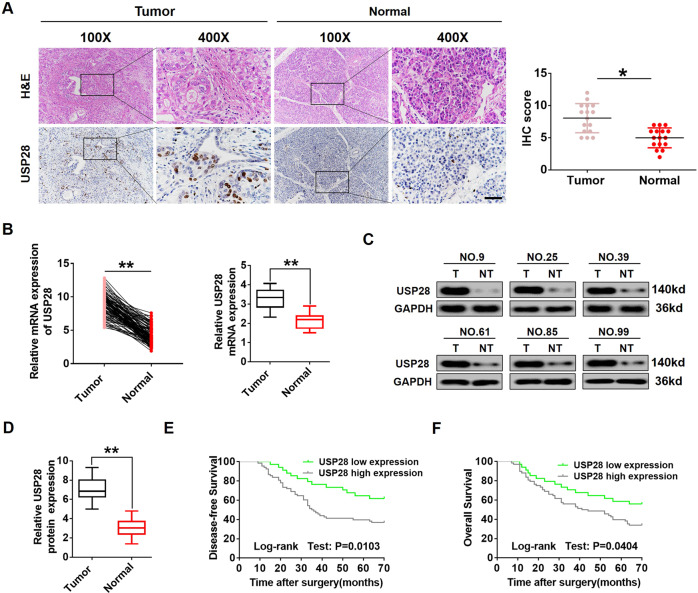
Overexpression of USP28 is correlated with poor prognosis in PC patients. **A** Representative H&E and IHC staining of USP28 in PC tissues and paired normal tissues (magnification ×100, inset magnification ×400). Scale bar, 50 μm. **B** qRT-PCR analysis of USP28 mRNA expression in 102 PC tumours and paired normal tissues. **P < 0.01. **C**, **D** Determination and quantification of USP28 protein levels in PC tissues and paired normal tissues by western blotting assay. GAPDH was used as a loading control. **P < 0.01. **E**, **F** Kaplan–Meier plots representing probabilities of progression-free and overall survival in 102 PC patients according to the expression level of USP28. Statistical analysis was conducted using Student *t* test and log-rank test.

To explore the role of USP28 in PC development, we examined USP28 expression in 102 PC tissue specimens and their corresponding normal tissues. As shown in Fig. 1A, immunohistochemistry (IHC) results revealed that USP28 was overexpressed in 66.7% (68/102) of the PC tissue specimens. Furthermore, qRT-PCR data indicated that *USP28* mRNA expression was significantly increased in PC tissues compared with that in normal tissues (Fig. 1B). In keeping with the increased USP28 mRNA, the protein level of USP28 was obviously higher in most cases (Fig. 1C, D). These results suggest that the expression of USP28 was up-regulated in human PC tissues.

Then, we assessed the association between USP28 expression and clinicopathological factors in 102 patients with PC (Table 1). The results indicated that there was no significant association between USP28 expression and age, histological type, or lymph node metastasis; however, it had a significant correlation with tumour size (*P* = 0.017), TNM stage (*P* < 0.001) and differentiation (*P* < 0.001). In addition, multivariate Cox regression analysis demonstrated that high USP28 level was an independent prognostic factor for poor survival of patients with PC (Table 2). To investigate the efficiency of USP28 expression in predicting the survival of PC patients, we analysed the relationship between USP28 level and the survival of PC patients. As shown in Fig. 1E, F, Kaplan–Meier analysis showed that PC specimens with high USP28 expression were associated with significantly poorer disease-free survival and overall survival than those with low USP28 expression. Collectively, these findings indicated that USP28 can serve as a valuable new prognostic factor for human PC.

**Table 2:** Univariate and multivariate analyses of overall survival in PC patients.

Parameters	Univariate analysis			Multivariate analysis		
	HR	95% CI	*P* value	HR	95% CI	*P* value
Age (≥65 vs <65)	1.764	0.721–2.986	0.876	—	—	—
Sex (female vs male)	1.636	0.584–2.927	0.618	—	—	—
Distant metastasis (no vs yes)	1.548	0.716–3.765	0.521	—	—	—
Histologic grade (high vs low to medium)	0.783	0.654–1.867	0.612	—	—	—
Tumour size (<5 vs ≥6)	1.687	1.153–5.176	0.006*	1.401	1.119–2.767	0.019*
TNM stage (T1–T2 vs T3-–T4)	2.577	1.475–5.242	0.026*	1.527	1.231–4.434	0.037*
Differentiation (well vs moderate/poor)	1.984	1.652–4.16	0.005*	1.211	0.933–3.655	0.237
USP28 expression (high vs low)	4.237	2.346–5.729	0.001*	2.125	1.456–4.217	0.023*

*HR* hazard ratio, *CI* confidence interval.

*P < 0.05.

### USP28 promotes PC cell growth in vitro and tumorigenesis in vivo

**Fig. 2:**
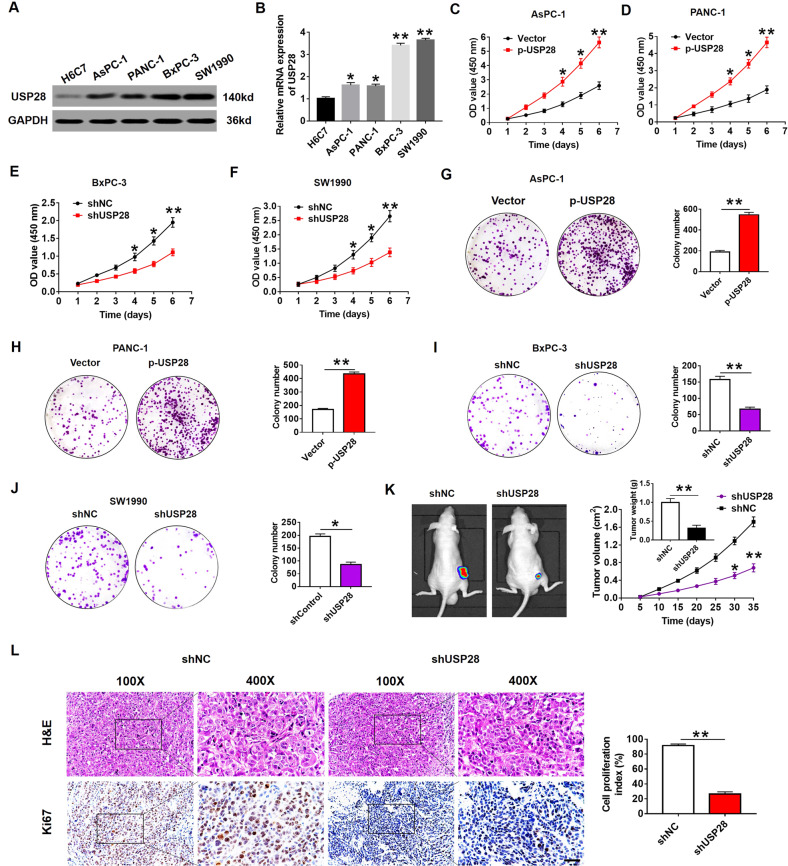
Effects of USP28 on PC cell growth. **A**, **B** The protein and mRNA levels of USP28 in four PC cells and the immortalised H6c7 line. **C**–**F** CCK-8 assay showing proliferation of PC cells following overexpression (**C** and **D**) or knockdown (**E** and **F**) of USP28. Data represent mean ± SD of triplicate experiments and were statistically analysed with Student’s *t* test, *P < 0.05, **P < 0.01. **G**–**J** Representative images (left) and quantification (right) of colony formation assays of pancreatic cancer cells transfected with p-USP28 (**G** and **H**) or shUSP28 (**I** and **J**). The data represent mean ± SD from three independent experiments and were statistically analysed with Student’s *t* test, *P < 0.05, **P < 0.01. **K** Bx*P*C-3/shUSP28 cells were subcutaneously injected into nude mice, and tumour volumes were measured on the indicated days; at the experimental endpoint, tumours were dissected, photographed and weighed. *n* = 6, *P < 0.05, **P < 0.01. **L** Representative H&E staining in tumour tissues isolated from USP28-silencing and control nude mice. And, cell proliferation in tumour tissues isolated from USP28-silencing nude mice was determined by Ki67 staining. Cell proliferation index was quantified by counting the proportion of nuclear Ki67-positive cells (magnification ×100, inset magnification ×400). **P < 0.01. Scale bar, 50 μm.

Western blot and qRT-PCR analysis showed that USP28 was overexpressed in the four PC cell lines (AsPC-1, PANC-1, BxPC-3 and SW1990) when compared with its expression in the control (H6C7; Fig. 2A, B). The frequent overexpression of USP28 in PC tumours and cell lines prompted us to explore its oncogenic role in PC. We generated USP28-overexpression AsPC-1 and PANC-1 cells, which show the relatively low endogenous USP28 expression (Supplementary Fig. 1). Meanwhile, USP28-targeting shRNA vectors were used to knockdown USP28 in BxPC-3 and SW1990 cells, which show high endogenous USP28 expression (Supplementary Fig. 1). USP28-overexpression AsPC-1 and PANC-1 cells significantly promoted cell proliferation, as evidenced by CCK-8 (Fig. 2C, D). Conversely, USP28-silencing in BxPC-3 and SW1990 cells suppressed cell growth (Fig. 2E, F). Consistent with these findings, Colony formation assays also showed that overexpression of USP28 increased the cell proliferation of AsPC-1 and PANC-1 cells (Fig. 2G, H), whereas USP28 knockdown decreased the cell proliferation of BxPC-3 and SW1990 cells (Fig. 2I, J).

To evaluate the effect of USP28 on tumorigenesis in vivo, we performed sub-cutaneous xenograft assay in nude mice using USP28-knockdown BxPC-3 cells. After 5 weeks of growth, significant reduction in tumour growth and weight was observed (Fig. 2K); this was concomitant with reduced cell proliferation (Ki67) (Fig. 2L). Furthermore, USP28-overexpression PC cells resulted in significantly greater tumour growth and tumour weight, when compared to their respective controls (Supplementary Fig. 2). Taken together, these data indicated that USP28 plays a crucial oncogenic role in promoting PC cell growth and tumorigenicity.

### USP28 promotes PC cell growth by facilitating cell cycle progression and inhibiting apoptosis

**Fig. 3:**
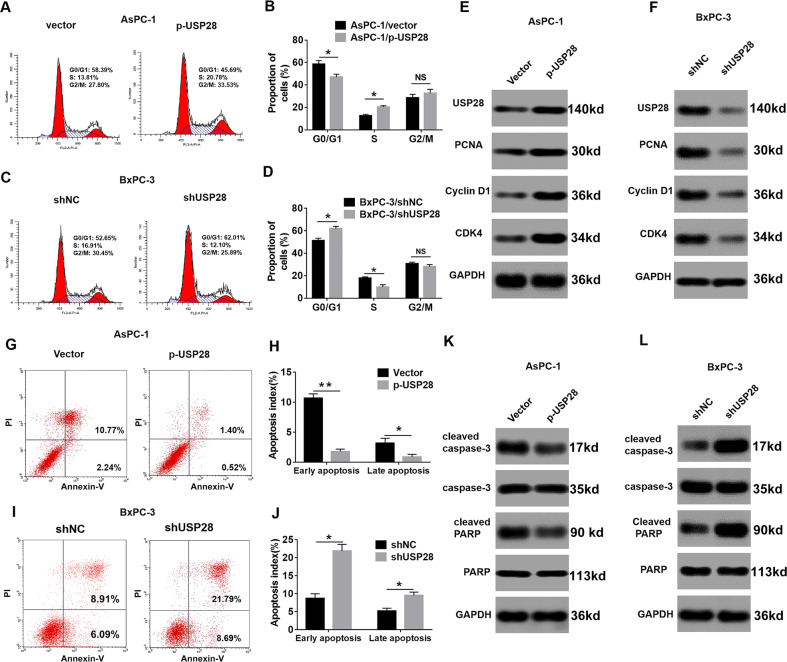
USP28 accelerates cell-cycle progression and inhibits cell apoptosis. **A**–**D** Detection for cell cycle of PC cells following overexpression or knockdown of USP28, respectively. Results are expressed as peak diagram (**A**, **C**) and calculated distribution for cells in G0/G1, S and G2/M phases (**B**, **D**). Data represent mean ± SD of triplicate experiments and were statistically analysed with Student’s *t* test, *P < 0.05. **E**, **F** Western blotting showing the protein expression of USP28, PCNA, cyclin D1 and CDK4 in USP28-overexpression AsPC-1 cells (**E**) or USP28-silencing BxPC-3 cells (**F**). GAPDH was used as a loading control. **G**, **H** Results are expressed as scatter diagram for measurement of apoptotic cells (**G**) and calculated percentage of annexin-V-positive cell population (**H**) in USP28-overexpression cells. The data represent mean ± SD from three independent experiments and were statistically analysed with Student’s *t* test, *P < 0.05, **P < 0.01. **I**, **J** Results are expressed as scatter diagram for measurement of apoptotic cells (**I**) and calculated percentage of annexin-V-positive cell population (**J**) in USP28-silencing cells. *P < 0.05. **K**, **L** Western blot for total and active form of caspase-3 and PARP in USP28-overexpression AsPC-1 cells (**K**) or USP28-silencing BxPC-3 cells (**L**). GAPDH was used as a loading control.

To investigate the mechanism by which USP28 contributes to PC cell growth, we examined the effect of USP28 on cell cycle progression and cell apoptosis. Ectopic expression of USP28 in AsPC-1 cells led to a significant reduction in G0/G1-phase cells and an increase in S-phase cells than in the control group (Fig. 3A, B). On the contrary, USP28 knockdown in BxPC-3 cells had an opposite effect on the cell cycle progression (Fig. 3C, D). Further, overexpression of USP28 enhanced the expression of proliferating cell nuclear antigen, cyclin D1, and cyclin-dependent kinase 4 (Fig. 3E), whereas USP28 knockdown inhibited the expression of these key cell cycle regulators (Fig. 3F). Moreover, the flow cytometry analysis showed that USP28 overexpression decreased both early and late apoptosis in AsPC-1 cells (Fig. 3G, H); whereas, USP28 knockdown in BxPC-3 cells increased apoptosis induction (Fig. 3I, J). The apoptosis suppression by USP28 was further evidenced by decreased protein expression of cleaved forms of caspase-3 and PARP in the USP28-overexpression cells (Fig. 3K); whereas, USP28-silencing showed enhanced expression of these apoptosis factors (Fig. 3L). Therefore, these results suggested that USP28 promotes PC cell growth via promotion of cell cycle progression and suppression of cell apoptosis.

### Wnt/β-catenin pathway acts as a downstream component of USP28 and contributes to the effects of USP28 in PC cells

**Fig. 4:**
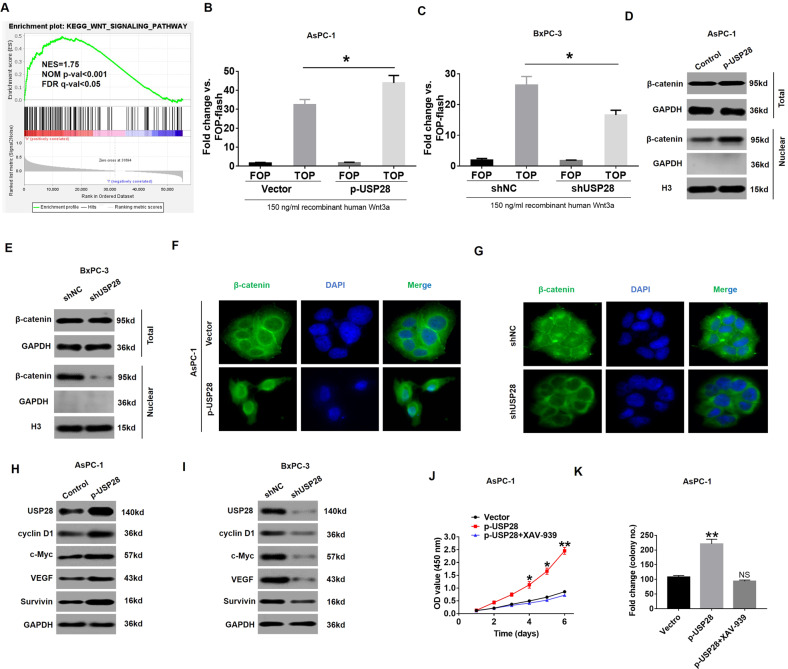
USP28 activates Wnt/β-catenin signalling pathway in PC cells. **A** GSEA comparing the gene sets of Wnt targets in USP28^high^ PC patients. Data were obtained from TCGA database. NES means normalized enrichment score. **B**, **C** After treatment with 150 ng/ml of recombinant human Wnt3a, the relative luciferase activity levels in cells transfected with TOP-flash and FOP-flash vectors in USP28-overexpression AsPC-1 cells (**B**) or USP28-silencing BxPC-3 cells (**C**) are shown. Data represent mean ± SD of triplicate experiments and were statistically analysed with Student’s *t* test, *P < 0.05. **D**, **E** The total and nuclear protein levels of β-catenin were assessed by western blotting in USP28-overexpression AsPC-1 cells (**D**) or USP28-silencing BxPC-3 cells (**E**). GAPDH and Histone 3 were used as a loading control, respectively. **F**, **G** IF analysis of the nuclear levels of the β-catenin in PC cell transduced with p-USP28 or shUSP28 plasmid. The green signal represents the staining of the corresponding protein, and the blue signal represents the nuclear DNA staining by DAPI. **H**, **I** Western blotting showing the protein expression of USP28, cyclin D1, c-Myc, VEGF and Survivin in USP28-overexpression AsPC-1 cells (**H**) or USP28-silencing BxPC-3 cells (**I**). GAPDH was used as a loading control. **J**, **K** Quantification for CCK-8 (**J**) or colony formation assays (**K**) of USP28-overexpression AsPC-1 cells transfected with XAV-939. *P < 0.05.

To explore the underlying mechanism of USP28-mediated tumorigenesis of PC, we firstly performed gene set enrichment analysis (GSEA) in TCGA database to explore the possible associations between USP28 and various signalling pathways. As shown Fig. 4A, the gene sets of Hallmark_Wnt_Targets were obviously enriched in PC samples with high USP28 level, indicating that the WNT pathway was closely associated with high USP28 level in PC. Then, we examined the activation of the Wnt/β-catenin signalling pathway after alternation of USP28 expression. As shown in Fig. 4B, C, after treatment with 150 ng/ml of recombinant human Wnt3a, we found that increased expression of USP28 in AsPC-1 cells significantly enhanced the activity of TOP-Flash reporter; whereas, USP28-silencing resulted in the decreased activity of the TOP-Flash reporter. In addition, knockdown of USP28 also inhibited the activation of the TOP-Flash reporter in BxPC-3 cells in the absence of recombinant human Wnt3a (supplementary Fig. 3). Importantly, compared with the control cells, the level of nuclear β-catenin was dramatically increased in PC cells with USP28 overexpression, and reduced in USP28-silenced cells (Fig. 4D, E). However, dysregulation of USP28 expression had no effect on the expression level of total β-catenin in PC cells (Fig. 4D, E). Consequently, the changes in nuclear β-catenin expression in USP28-overexpression and USP28-knockdown PC cells were further confirmed by immunofluorescence experiments (Fig. 4F, G). In addition, the expression of USP28 was indicated by the immunofluorescence images in USP28-overexpression and USP28-knockdown PC cells (Supplementary Fig. 4). Overexpression of USP28 also increased the expression of the Wnt/β-catenin target genes: cyclin-D1, c-Myc, VEGF and surviving (Fig. 4H), which are key genes related to cell proliferation and apoptosis [23–25]. In contrast, USP28-silencing led to reduced expression of these genes (Fig. 4I).

Next, we inhibited Wnt/β-catenin signalling pathway in USP28-overexpression cells by using XAV-939, a specific inhibitor of β-catenin [26]. Interestingly, the enhanced cell viability in PC cells caused by USP28 overexpression was significantly suppressed by XAV-939 (Fig. 4J, K). In addition, our data demonstrated that increased intensity c-Myc and cyclin-D1 in USP28-overexpression PC cells was remarkably abolished by XAV-939 (Supplementary Fig. 5). Collectively, these results suggested that the Wnt/β-catenin signalling pathway is critical for USP28-mediated oncogenic function in PC.

### USP28 activates Wnt/β-catenin signalling pathway by modulating FOXM1 expression

**Fig. 5:**
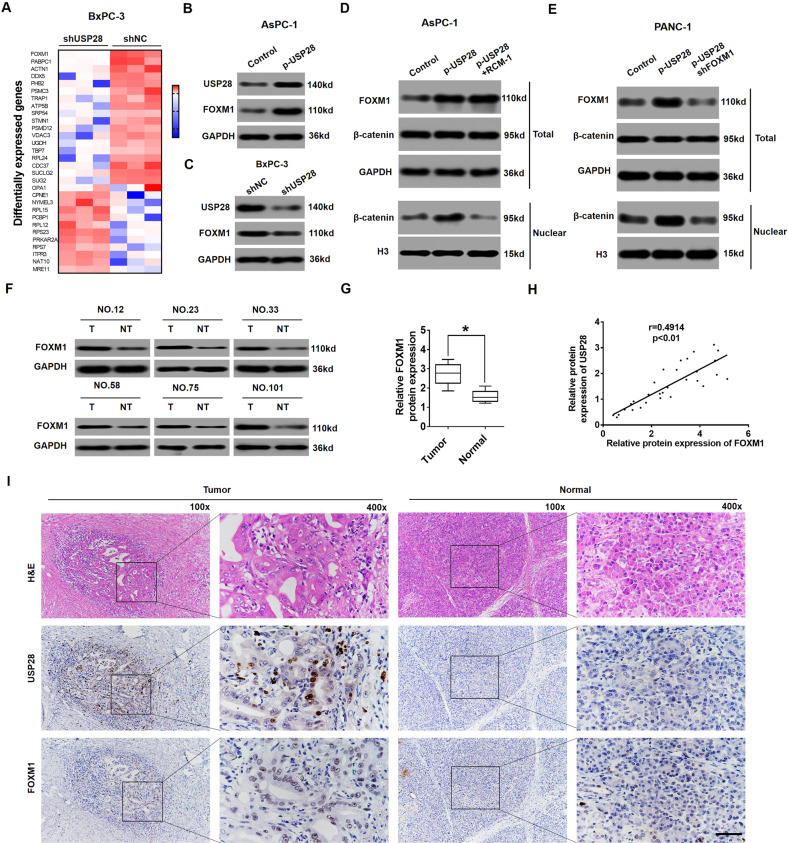
USP28 positively regulates FOXM1 protein levels in PC cells. **A** Heatmap of genes differentially expressed following USP28 depletion by mass spectroscopic analysis. **B**, **C** Western blot for USP28 and FOXM1 expression in USP28-overexpression AsPC-1 cells (**B**) or USP28-silencing BxPC-3 cells (**C**). GAPDH was used as a loading control. **D**, **E** The total and nuclear protein levels of β-catenin were assessed by western blotting in USP28-overexpression AsPC-1 cells following treatment with RCM-1 (**D**) or USP28-overexpression PANC-1 cells following treatment with shFOXM1 (**E**). GAPDH and Histone 3 were used as a loading control, respectively. **F**, **G** Determination (**F**) and quantification (**G**) of FOXM1 protein levels in PC tissues and in paired normal tissues by western blot assay. GADPH was a loading control. *P < 0.05. **H** Scatter plots show a positive correlation between USP28 and FOXM1 at the protein level in PC. **I** Representative IHC staining of USP28 and FOXM1 in PC tissues and paired normal tissues (magnification ×100, inset magnification ×400). Scale bar, 50 μm.

To determine the underlying mechanisms by which USP28 activates Wnt/β-catenin signalling pathway in PC, we performed large-scale proteomics experiments by TMT-based LC-MS/MS assay in USP28-silenced PC cells. We found that FOXM1 protein expression was significantly down-regulated in USP28-silenced PC cells (Fig. 5A). Further investigation revealed that the protein level of FOXM1 was significantly increased in USP28-overexpression cells, and reduced in USP28-knockdown cells, thereby indicating that USP28 positively regulates FOXM1 level (Fig. 5B, C). Importantly, it has been reported that FOXM1 enhances β-catenin nuclear-localisation and transcriptional activity in PC [27]. Thus, we speculated that USP28 activates Wnt/β-catenin signalling pathway by enhancing FOXM1 expression in PC. As expected, the level of nuclear β-catenin was significantly increased by USP28 overexpression and reduced by Robert Costa Memorial drug-1 [28], which is a small-molecule inhibitor of FOXM1 (Fig. 5D). FOXM1-silencing also suppressed the level of nuclear β-catenin in USP28-overexpression PC cells (Fig. 5E), indicating that FOXM1 was responsible for USP28-mediated activation of Wnt/β-catenin signalling pathway. In addition, the increased intensity of nuclear β-catenin in USP28-overexpression PC cells was reversed by inhibition of FOXM1 (Supplementary Fig. 6).

To further confirm the relationship between USP28 and FOXM1, we examined the expression of FOXM1 in 102 PC specimens with high USP28 expression by western blot assay. As a result, 76 cases (74.5%) showed significant upregulation of FOXM1 in PC tissues compared with FOXM1 expression in normal pancreatic tissues (Fig. 5F, G). Importantly, the statistical analysis data indicate that the protein level of FOXM1 was positively correlated with the protein level of USP28 in PC tissues (Fig. 5H). Finally, IHC data confirmed that the levels of USP28 and FOXM1 were both elevated in the PC tissues (Fig. 5I), which was consistent with the western blot results. Overall, these findings suggested that FOXM1 may mediate the regulatory function of USP28 in PC cells.

### USP28 interacts with FOXM1 and stabilises FOXM1 expression

**Fig. 6:**
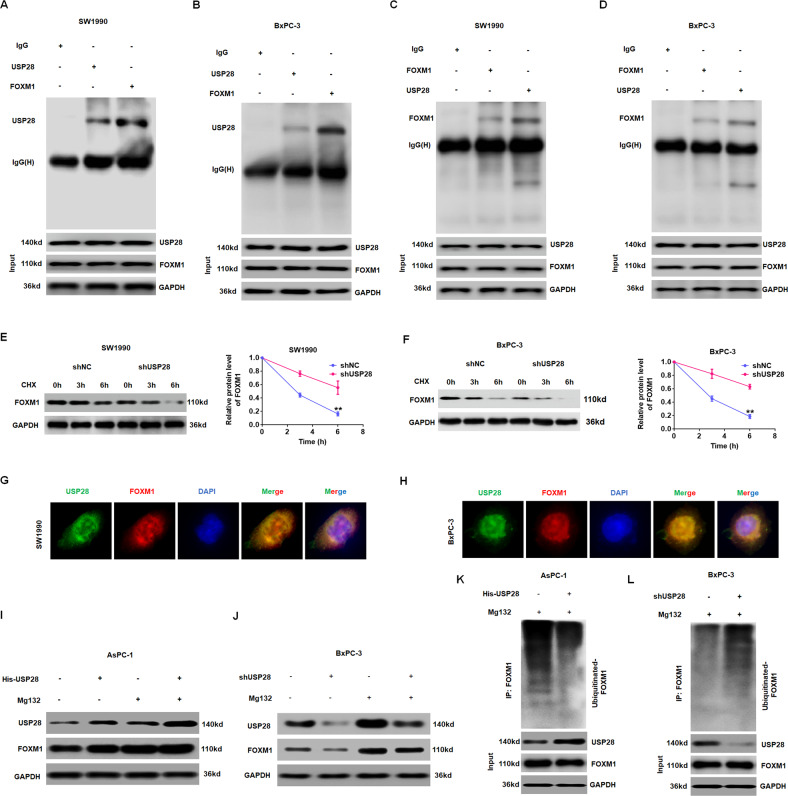
USP28 interacting with FOXM1 and stabilising FOXM1 expression via deubiquitination. **A**–**D** The interaction between USP28 and FOXM1 was confirmed by co-immunoprecipitation (IP) in SW1990 cells and BxPC-3 cells. **E**, **F** Co-localisation studies of PC cells using anti-USP28 antibody (1:100, green) and anti-FOXM1 antibody (1:100, red), followed by DAPI nuclear counterstaining (blue). The merged images of USP28 (green) and FOXM1 (red) with DAPI (blue) are also shown. Scale bar, 50 μm. **G**, **H** Representative (right) and quantitative (left) results of FOXM1 protein level in USP28-silencing cells. The cells were treated with cycloheximide (CHX, 100 mg/ml) for indicated time points were subjected to western blot analysis. **P < 0.01. **I** J PC cells transduced with p-USP28 (**I**) or shUSP28 (**J**) were treated with 10 μM MG132. Cells were collected at 6 h and immunoblotted with the antibodies indicated. **K**, **L** Lysates from PC cells transduced with p-USP28 (**K**) or shUSP28 (**L**) were immunoprecipitated with the anti-Ub and immunoblotted with the anti-FOXM1. Cells were treated with MG132 for 6 h before collection.

We further explored the mechanism by which USP28 regulates FOXM1 expression in PC. Our data demonstrated that the mRNA level of *FOXM1* was not significantly different after alteration of USP28 in PC cells (Supplementary Fig. 7), indicating that USP28 positively regulates FOXM1 at post-transcriptional level. In addition, studies have shown that FOXM1 undergoes degradation by ubiquitin-protease system [29, 30]. Given that USP28 is a deubiquitinase, we hypothesised that USP28 may regulate FOXM1 ubiquitination and degradation in PC. To test this hypothesis, we first examined whether USP28 interacted with FOXM1 in PC cells. As shown in Fig. 6A–D, the Co-IP assays confirmed the interaction between USP28 and FOXM1 using endogenous USP28 and FOXM1 antibodies in SW1990 and BxPC-3 cells. Further, confocal assays further demonstrated that co-localisation of USP28 and FOXM1 was significantly evident in PC cells (Fig. 6E, F). Secondly, we assessed the degeneration of FOXM1 protein in USP28-knockdown cells after cycloheximide exposure. As shown in Fig. 6G, H, the data indicate that USP28-silencing significantly promoted the degeneration of FOXM1 protein in PC cells. Thirdly, we found that the protein level of FOXM1 was restored in USP28-overexpression or USP28 knockdown PC cells following treatment with proteasome inhibitor MG132 (Fig. 6I, J). Finally, ectopic expression of USP28 decreased the ubiquitination level of FOXM1, whereas knockdown of USP28 increased the poly-ubiquitination of FOXM1 (Fig. 6K, L). Therefore, these findings suggested that USP28 serves as a deubiquitinase that is responsible for FOXM1 stabilisation via the ubiquitin-proteasome pathway in PC.

### USP28 promotes PC progression via enhancing FOXM1 levels

**Fig. 7:**
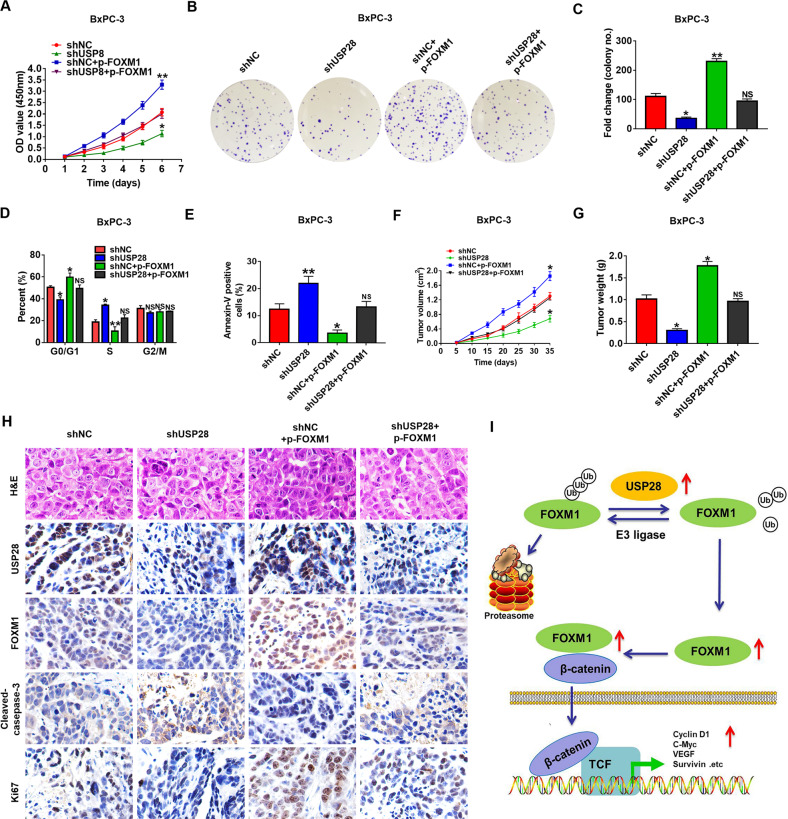
Oncogenic effect of USP28 is dependent on FOXM1 stabilisation. **A**–**C** Restoration of FOXM1 expression impaired the anti-tumour effect caused by USP28 knockdown, as determined by CCK-8 (**A**) and colony formation assay (**B** and **C**) in AsPC-1 cells. **D**, **E** The quantification of cell cycle (**D**) or cell apoptosis (**E**) assay in the different groups. The data represent mean ± SD from three independent experiments and were statistically analysed with Student’s *t* test, *P < 0.05, **P < 0.01. **F**, **G** The quantification of tumour volume (**F**) or tumour weight (**G**) assay in the different groups. *P < 0.05. **H** Representative H&E and IHC staining of USP28, FOXM1, Cleaved-caspase-3 and Ki67 in tumour tissues isolated from different nude mice groups. Scale bar, 50 μm. **I** Proposed mechanistic scheme of USP28 in promoting the FOXM1-mediated Wnt/β-catenin signalling pathway in pancreatic cancer.

Lastly, we performed rescue experiments to determine whether the oncogenic effect of USP28 in PC is dependent on FOXM1 stabilisation. As shown in Fig. 7A–C, USP28 knockdown markedly decreased cell proliferation in PC cells, which were attenuated by concomitant overexpression of FOXM1. Consistently, FOXM1 enhancement rescued cell cycle arrest in G0/G1 phase induced by USP28 knockdown in PC cells (Fig. 7D). Meanwhile, flow cytometry assays revealed that the pro-apoptotic effect of USP28 knockdown could be partially reversed by the introduction of FOXM1 in PC cells (Fig. 7E). Moreover, we subcutaneously implanted PC cells with either USP28 single-knockdown or USP28 knockdown with FOXM1 overexpression into nude mice and monitored their tumour growth. As shown in Fig. 7F, G, mice bearing USP28-silenced cells showed a notable decrease in tumour weight and volume; whereas, simultaneous overexpression of FOXM1 fully abolished the anti-tumour effect of USP28-knockdown. In line with these data, the IHC assays revealed increased intensity cleaved-caspase-3, and reduced FOXM1 expression and Ki67-positive proliferative cells in USP28-knockdown tumours. In contrast, concomitant overexpression of FOXM1 reversed such effects (Fig. 7H). Therefore, USP28 promoted the tumorigenesis of PC progression by stabilising FOXM1.

## Discussion

In this study, we demonstrated that USP28 was highly expressed in PC tissues and increased expression of USP28 was closely correlated with malignant phenotype and survival probability of patients with PC. In addition, our study examined the role of USP28 in PC tumorigenesis using cell culture approaches and animal-based tumour models. The deubiquitinase USP28 has been identified as an oncogene that plays critical roles in tumorigenesis in diverse cancer types [8, 11]. However, no information is currently available on the role or molecular mechanism of USP28 in PC, and we believe that this is to the best of our knowledge the first study to explore the role of USP28 in PC. We found that USP28 promoted the proliferation, apoptosis, and tumorigenesis of PC cells, thereby indicating that USP28 may serve as an oncogene in PC.

Wnt/β-catenin signalling is an evolutionarily conserved and versatile pathway that is involved in carcinogenesis and tumour progression of several cancers [31, 32]. β-catenin is a key component of the Wnt/β-catenin signalling pathway and it is tightly regulated at three hierarchical levels: protein stability, sub-cellular localisation, and transcriptional activity [33, 34]. Studies have shown that β-catenin protein levels increase in PC tissues, and high level of nuclear-localised β-catenin is closely associated with poor clinical outcomes in patients with PC [35–37]. This study identified that USP28 increased the nuclear translocation of β-catenin, luciferase reporter activity of β-catenin, and expression of the target proteins of the Wnt/β-catenin pathway. In addition, inhibitor of β-catenin (XAV-939) reversed PC cell viability that was induced by USP28. Therefore, these results suggest that USP28 contributes to PC progression by promoting nucleus β-catenin transactivation and transcriptional activity.

FOXM1 is a critical mediator of Wnt/β-catenin signalling and acts by binding to β-catenin to enhance β-catenin nuclear-localisation and downstream target gene expression [27, 38]. However, the role of the upstream regulators of FOXM1 expression remain unclear. Recent evidence has demonstrated that post-translational modifications, especially ubiquitination, play a pivotal role in FOXM1 regulation [29, 39, 40]. While FOXM1 can undergo ubiquitination and degradation mediated by various E3-ubiquitin ligases [41, 42], it remains unclear how FOXM1 is regulated by deubiquitination pathways. In the current study, we identified USP28 as a specific deubiquitinase for FOXM1 in PC cells. USP28 has been previously reported to regulate ubiquitination of other substrates as well in diverse cancers. In accordance with previous studies, we showed that USP28 directly interacted with FOXM1 and suppressed the poly-ubiquitination of FOXM1, thereby stabilising FOXM1 expression in PC cells. In addition, we observed that the oncogenic effect of USP28 in PC was dependent on FOXM1 stabilisation. Although the broader role of USP28 in regulating other targets in cancers remains to be determined, identification of USP28 as a FOXM1 deubiquitinase will hopefully lead to additional studies focused on USP28 as a therapeutic target in PC.

## Conclusions

In this study, we identified that USP28 served as a deubiquitinase for FOXM1 in PC. USP28 interacted with FOXM1 and reduced the poly-ubiquitination of FOXM1, thereby stabilising FOXM1 and leading to the activation of the Wnt/β-catenin pathway. Further, high USP28 level was closely associated with progression and poor outcome of patients with PC. Furthermore, deletion of USP28 inhibited the growth of PC cells both in vitro and in vivo. Taken together, these results suggest that USP28 contributes to PC pathogenesis via enhancing the FOXM1-mediated Wnt/β-catenin signalling (Fig. 7I), thereby indicating that targeting USP28/FOXM1/β-catenin axis could be a potential strategy for PC therapy.

## Supplementary information

Supplementary Figure 1

Supplementary Figure 2

Supplementary Figure 3

Supplementary Figure 4

Supplementary Figure 5

Supplementary Figure 6

Supplementary Figure 7

Supplementary Figure legends

## Data Availability

All data generated or analysed during this study are included in this published article. Additional datasets used and/or analysed during the current study are available from the corresponding author on reasonable request.
