# Supplementary figures and images for: USP28 facilitates pancreatic cancer progression through activation of Wnt/β-catenin pathway via stabilising FOXM1
Source: Cell Death Dis. 2021 Sep 28;12(10):887. doi: 10.1038/s41419-021-04163-z (PMC8478945; doi:10.1038/s41419-021-04163-z)

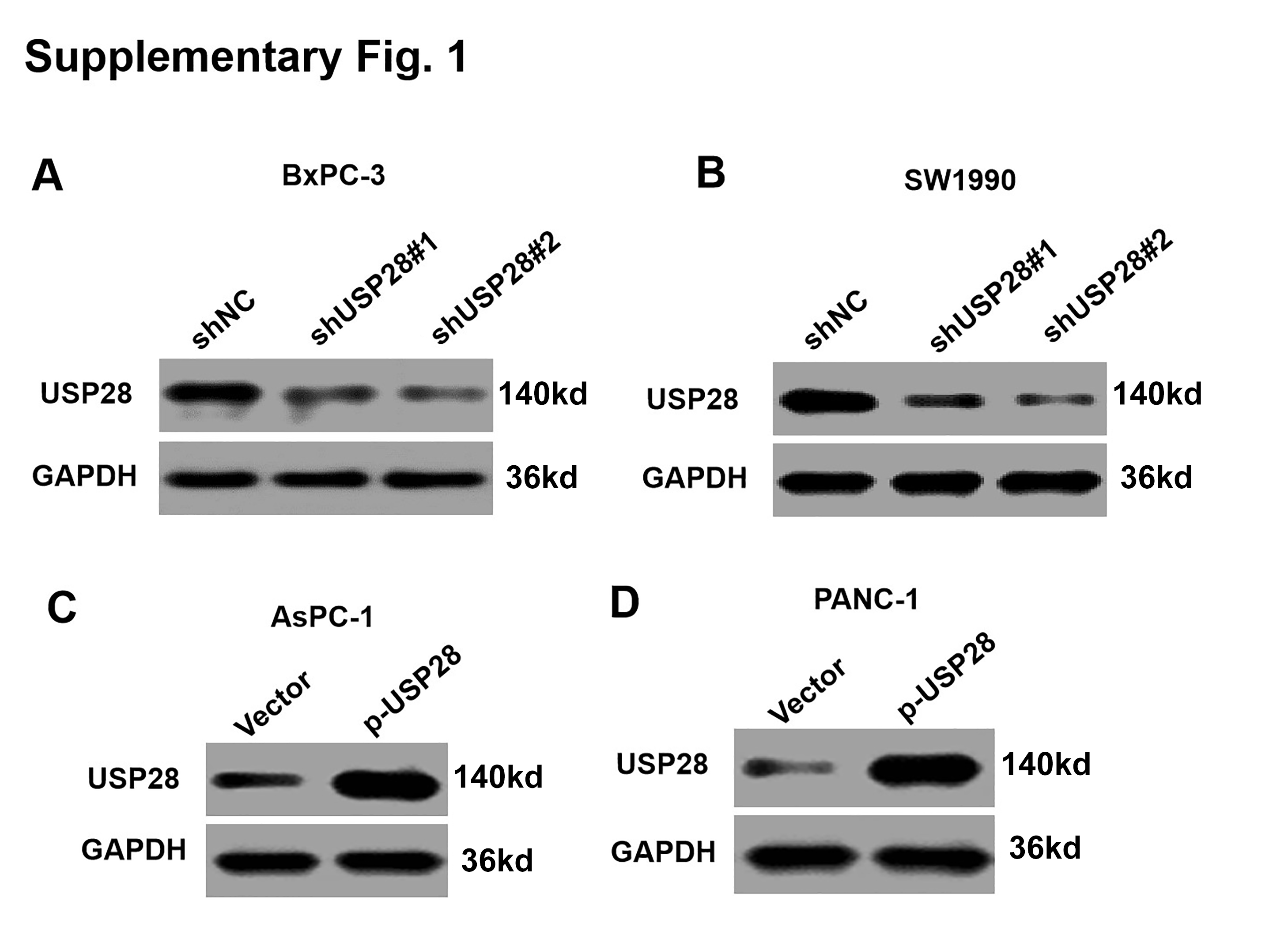

Supplement: Supplementary file 1 — Supplementary Figure 1 [file 41419_2021_4163_MOESM1_ESM.tif]

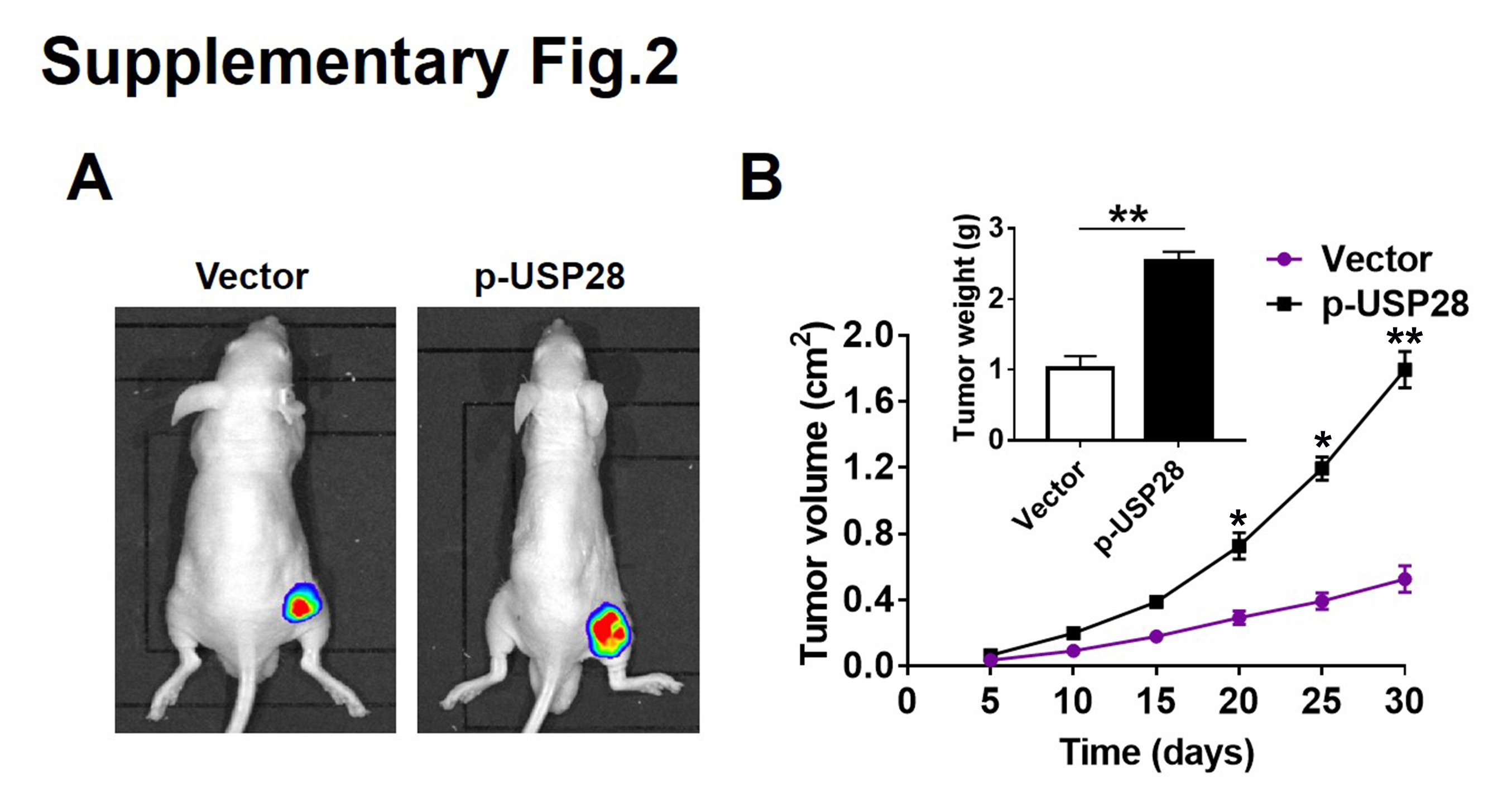

Supplement: Supplementary file 2 — Supplementary Figure 2 [file 41419_2021_4163_MOESM2_ESM.tif]

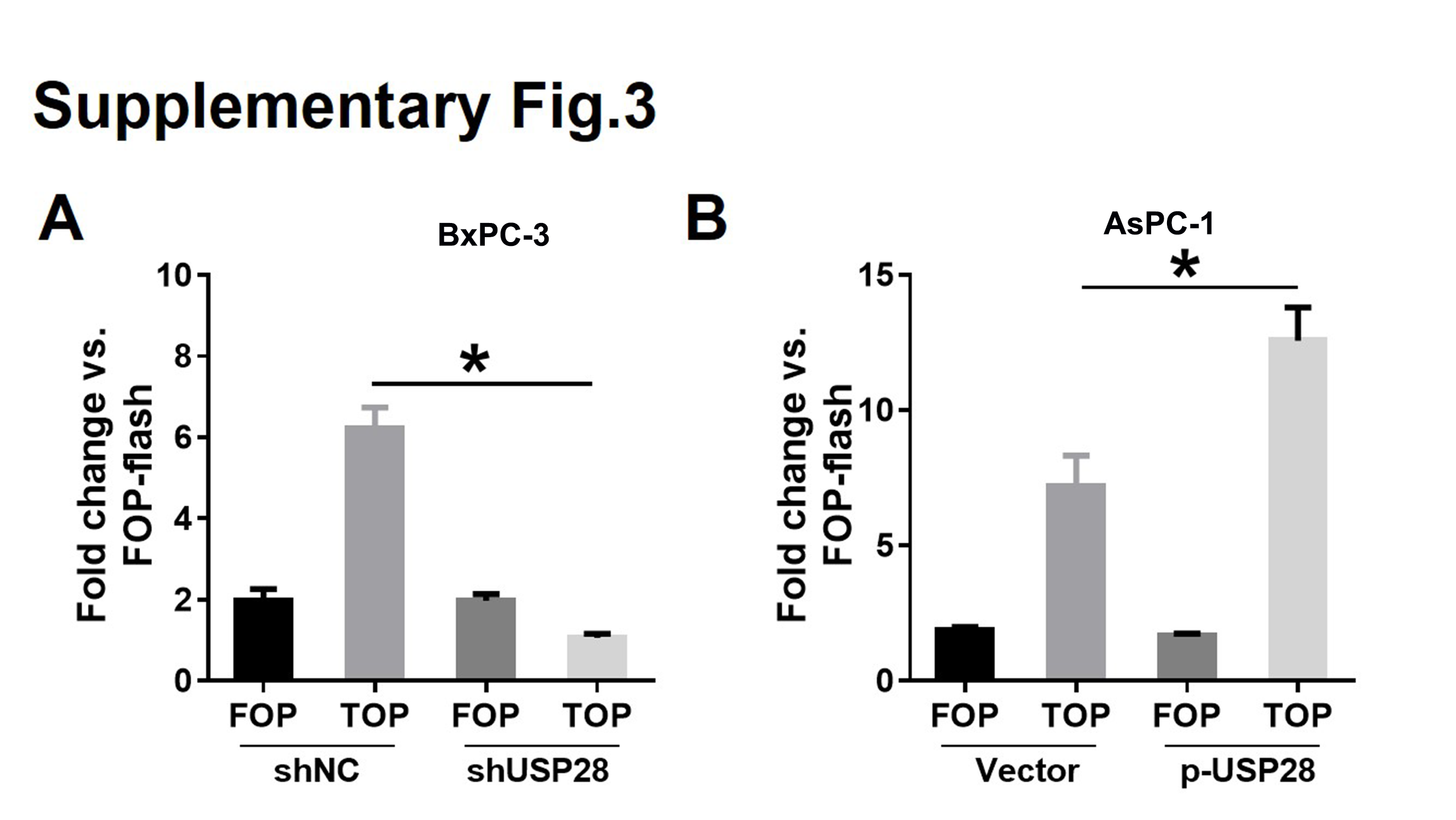

Supplement: Supplementary file 3 — Supplementary Figure 3 [file 41419_2021_4163_MOESM3_ESM.tif]

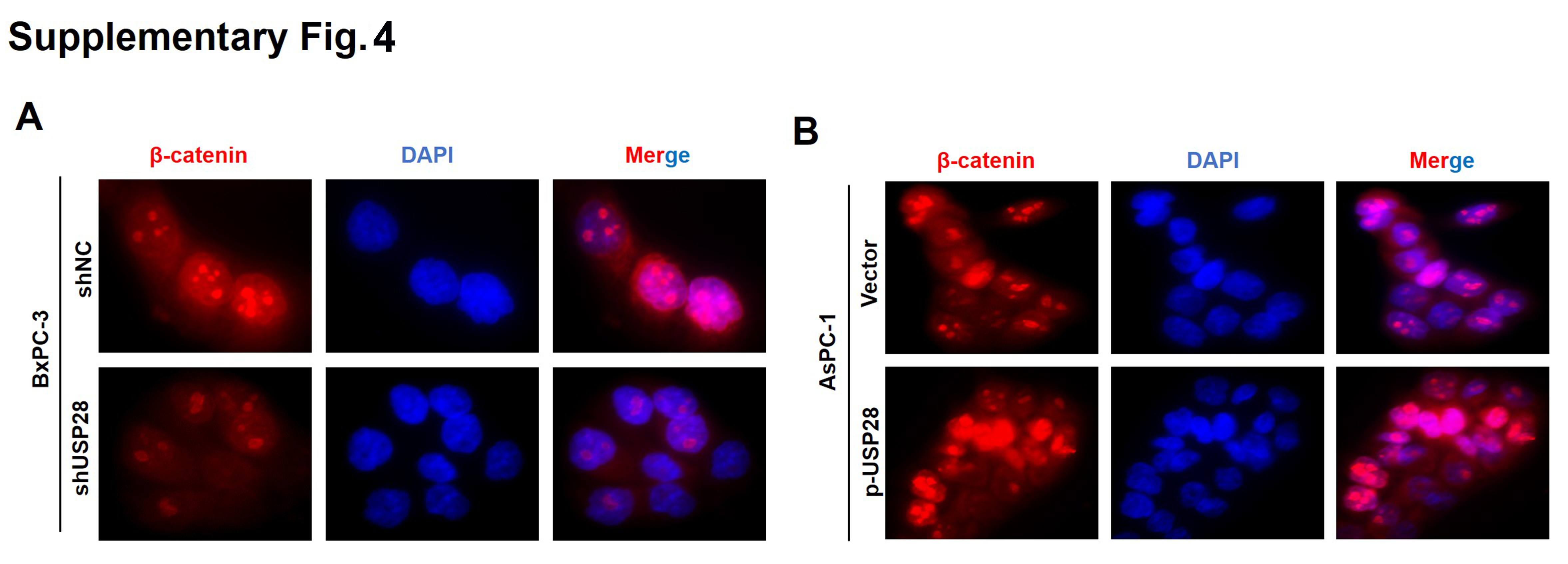

Supplement: Supplementary file 4 — Supplementary Figure 4 [file 41419_2021_4163_MOESM4_ESM.tif]

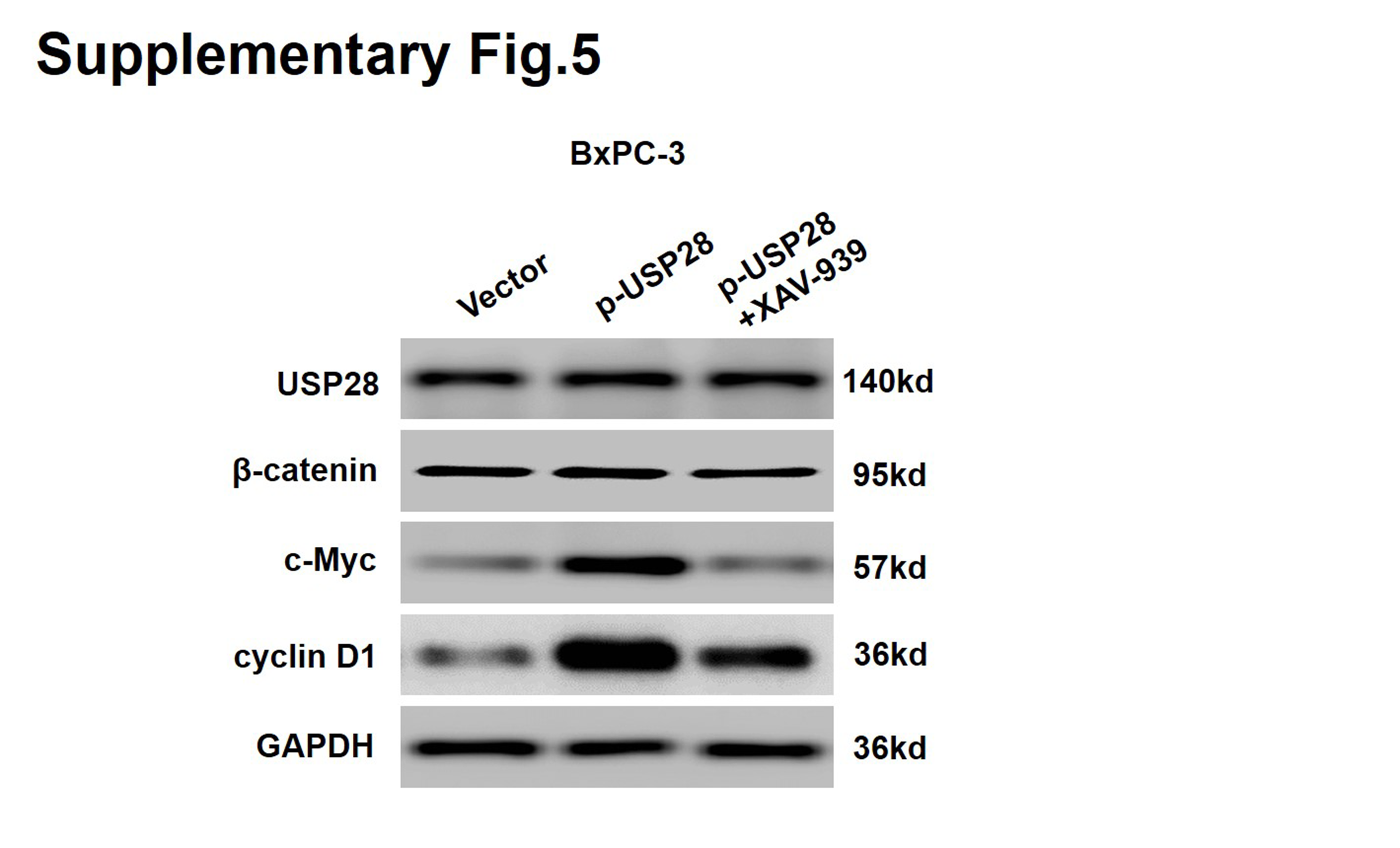

Supplement: Supplementary file 5 — Supplementary Figure 5 [file 41419_2021_4163_MOESM5_ESM.tif]

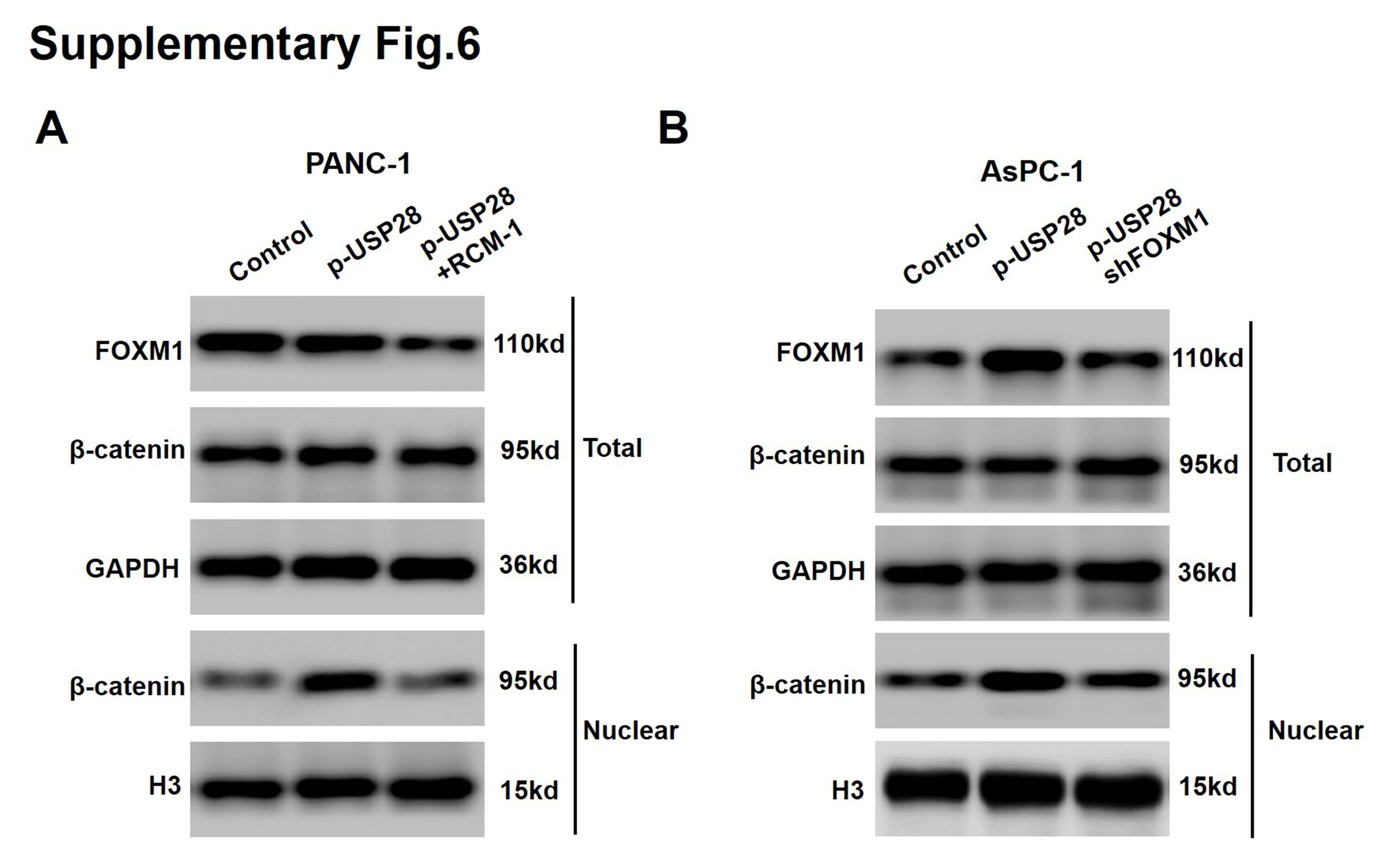

Supplement: Supplementary file 6 — Supplementary Figure 6 [file 41419_2021_4163_MOESM6_ESM.tif]

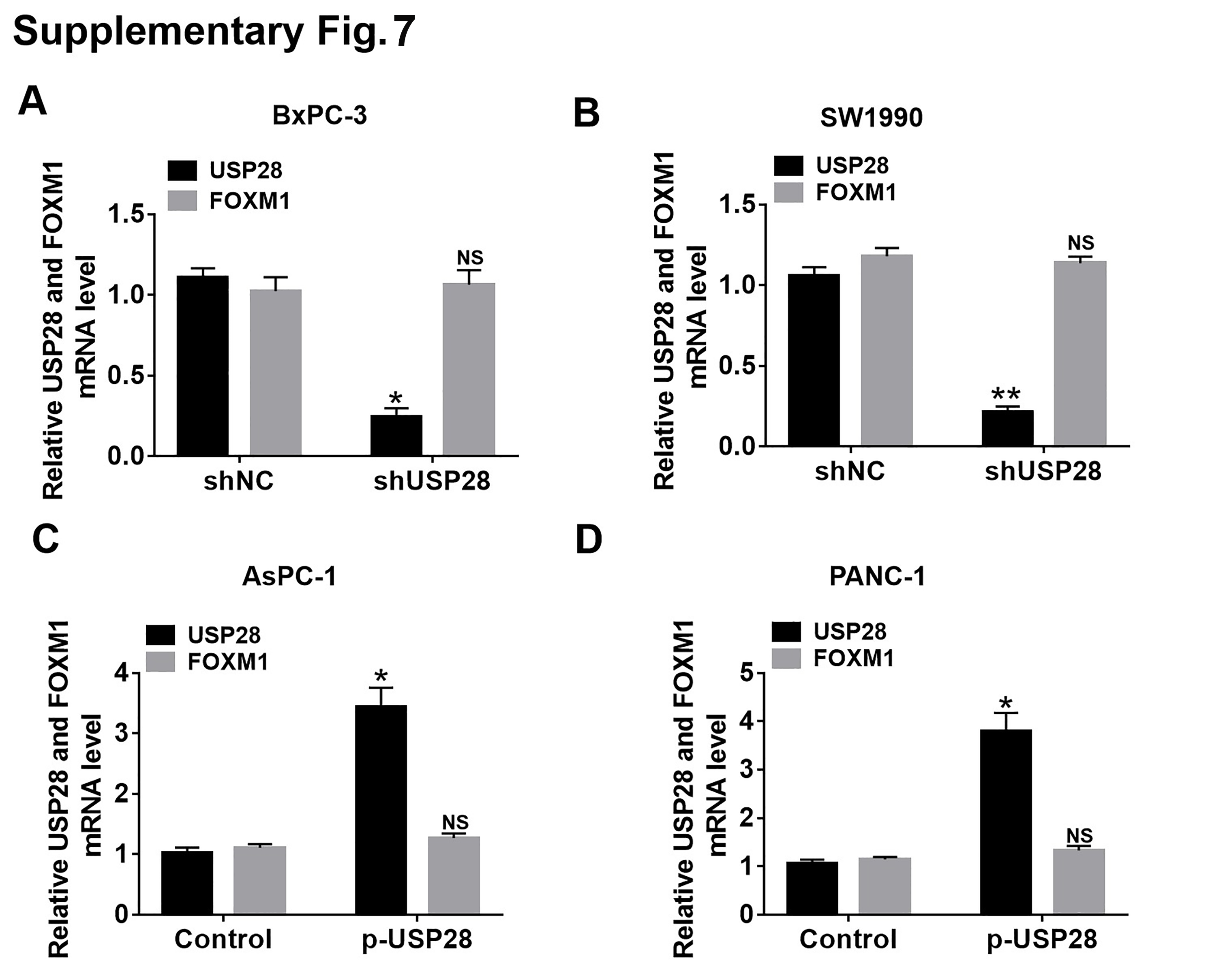

Supplement: Supplementary file 7 — Supplementary Figure 7 [file 41419_2021_4163_MOESM7_ESM.tif]
