# Supplementary material for: USP28 facilitates pancreatic cancer progression through activation of Wnt/β-catenin pathway via stabilising FOXM1
Source: Cell Death Dis. 2021 Sep 28;12(10):887. doi: 10.1038/s41419-021-04163-z (PMC8478945; doi:10.1038/s41419-021-04163-z)
Supplement: Supplementary file 8 — Supplementary Figure legends [file 41419_2021_4163_MOESM8_ESM.doc]

**Supplementary Figure legends**

**Supplementary Figure 1. Efficiency of USP28 interference or overexpression in PC cells.** A and B. Protein levels of USP28 were assessed in BxPC-3 and SW1990 cells transfected with shNC or shUSP28 by western blotting assay. GAPDH was used as a loading control. C and D. Protein levels of USP28 assessed in AsPC-1 and PANC-1 cells transfected with vector or p-USP28 plasmid by western blotting assay. GAPDH was used as a loading control.

**Supplementary Figure 2. Effects of USP28 overexpression on PC cell growth.** A and B, AsPC-1/p-USP28 cells were subcutaneously injected into nude mice, and tumour volumes were measured on the indicated days; at the experimental endpoint, tumours were dissected, photographed, and weighed. n=6, ^*^*P*<0.05, ^**^*P*<0.01.

**Supplementary Figure 3. Dual-luciferase reporter assay in USP28-over-expression or USP28-knockdown PC cells.** A and B, USP28-knockdown BxPC-3 cells (A) or USP28-over-expression AsPC-1 cells (B) were transfected with the TOP/FOP-Flash reporter plasmid, and the reporter activities were detected 48 h after transfection by a luciferase assay. **P*<0.05.

**Supplementary Figure 4. Efficiency of USP28 interference or overexpression in PC cells was tested by IF analysis.** A and B, the nuclear levels of the USP28 in PC cell transduced with p-USP28 or shUSP28 plasmid. The red signal represents the staining of the corresponding protein, and the blue signal represents the nuclear DNA staining by DAPI.

**Supplementary Figure 5.** Western blotting showing the protein expression of USP28, β-catenin, c-Myc and cyclin D1 in USP28-overexpression AsPC-1 cells transfected with XAV-939. GAPDH was used as a loading control.

**Supplementary Figure 6. Western blotting showing the protein expression of FOXM1 and β-catenin.** A and B, the total and nuclear protein levels of β-catenin were assessed by Western blotting in USP28-overexpression PC cells following treatment with RCM-1 (A) or shFOXM1 (B). GAPDH and Histone 3 were used as a loading control, respectively.

**Supplementary Figure 7. The mRNA levels of USP28 and FOXM1 were detected.** A and B, The mRNA levels of USP28 and FOXM1 assessed by qRT-PCR in PC cells transfected with shNC or shUSP28. C and D, The mRNA levels of USP28 and FOXM1 assessed by qRT-PCR in PC cells transfected with vector or p-USP28 plasmid.
